# Association between IL-10 Gene Polymorphisms and Susceptibility of Tuberculosis: Evidence Based on a Meta-Analysis

**DOI:** 10.1371/journal.pone.0088448

**Published:** 2014-02-11

**Authors:** Bin Liang, Yang Guo, Yunhui Li, Hong Kong

**Affiliations:** 1 Department of Clinical Laboratory, High Vocational Technological College, China Medical University, Shenyang, China; 2 Department of Breast Surgery, General Surgery, The First Hospital of China Medical University, Shenyang, China; 3 Department of Clinical Laboratory, No.202 Hospital, Shenyang, China; 4 Department of Clinical Laboratory, Shengjing Hospital, China Medical University, Shenyang, China; Archivel Farma; Fundació Institut d’Investigació en Ciències de la Salut Germans Trias i Pujol. Universitat Autònoma de Barcelona. CIBERES, Spain

## Abstract

**Background:**

A number of observational studies have been conducted to investigate the association of IL-10 gene polymorphisms with tuberculosis (TB) susceptibility. However, the results of different studies were inconsistent. The aim of this study was to investigate the relationship between IL-10 -1082G/A, -819T/C, and -592A/C polymorphisms and TB risk by meta-analysis.

**Methods:**

A literature search was conducted among six English databases (PubMed, Embase, Web of Science, Science Direct, SpringerLink and EBSCO) and two Chinese databases (Wanfang and Chinese National Knowledge Infrastructure databases) to identify studies involving association between IL-10 −1082G/A, −819T/C, and −592A/C polymorphisms and TB susceptibility before May. 2013. Statistical analysis was performed using Revman 5.0 and Stata 12.0.

**Results:**

A total of 31 studies with 6,559 cases and 7,768 controls were included in this meta-analysis. The results showed that three polymorphisms (-1082G/A, -819T/C, and -592A/C) in the IL-10 gene were not associated with the risk of TB in general population. In the subgroup analysis by ethnicity, IL-10 -1082G/A polymorphism was associated with TB risk in Europeans (AA+AG vs. GG: OR =  0.57, 95% CI = 0. 0.37–0.89, *P* = 0.01) and Americans (AA+AG vs. GG: OR =  0.39, 95% CI = 0.27–0.57, *P*<0.01), and IL-10 -819T/C (C allele vs. T allele: OR = 0.83, 95% CI = 0.72–0.96, *P* = 0.01) and -592A/C (CC+AC vs. AA: OR =  0.65, 95% CI = 0.49–0.85, *P* = 0.002) polymorphisms were significantly associated with TB risk in Asians.

**Conclusion:**

This meta-analysis provides strong evidence that IL-10-1082G/A polymorphism was associated with TB risk in Europeans and Americans, and IL-10 -819T/C and -592A/C polymorphisms could be risk factors for TB in Asians. Additional well designed large studies were required for the validation of our results.

## Introduction

Tuberculosis (TB) is a chronic infectious disease that occurs worldwide, leading to 1.6 million deaths annually worldwide [1]. However, from the estimated 2 billion individuals that have been initially infected with *Mycobacterium tuberculosis (M. tuberculosis)*, only 5% to 10% develop symptomatic TB [2]. The exact reasons as to why only some of the individuals exposed to *M. tuberculosis* develop uncontrolled disease and others have an effective immune response to limit the spread of the pathogen remains unknown. The genetic influence on TB infection was established by several studies of monozygotic and dizygotic twins, linkage and candidate gene analysis, indicating that genetics may play a role in the susceptibility to TB infection [3]–[5].

A major determinant for the clinical expression of the different forms of TB, and their final outcome, is the interaction between the pathogen and the host immune system. Cytokines play an important role in anti-TB immune response, and cytokines interleukin -10 (IL-10) have been implicated in the pathogenesis of TB [6]. IL-10 is an important immunoregulatory cytokine mainly produced by macrophages, monocytes, T cells, B cells, dendritic cells, mast cells and eosinophils [7]. Turner J, *et al.* demonstrated that increased susceptibility to reactivation tuberculosis in the mouse model is strongly influenced by the expression of IL-10 during the chronic or latent phase of the infection [8]. IL-10 potentially helps *M. tuberculosis* persistence in humans by blocking phagosome maturation in macrophages [9]. The ability of IL-10 to down-regulate immune responses and the fact that IL-10 can be detected in tuberculosis patients have led researchers to investigate whether IL-10 plays a role in susceptibility to tuberculosis [10], [11].

Certain single nucleotide polymorphisms within the promoter region of the IL-10 gene have been associated with altered levels of circulating IL-10, such as −1082G/A, −819T/C, and −592A/C [12], [13]. These polymorphisms have been investigated as potential susceptibility factors for TB. Given the functional significance of this genetic variant, a number of case-control studies have been done in different populations to investigate its susceptibility towards TB, but the findings remain conflicting rather than conclusive. Therefore, we conducted a systematic review and meta-analysis to get a more precise estimate of the association between IL-10 polymorphisms and TB risk.

## Materials and Methods

### Literature Search Strategy

A literature search was conducted among six English databases (PubMed, Embase, Web of Science, Science Direct, SpringerLink and EBSCO) and two Chinese databases (Wanfang and Chinese National Knowledge Infrastructure databases) to identify studies involving association between IL-10 −1082G/A, −819T/C, and −592A/C polymorphisms and TB susceptibility before May. 2013. Key words used in the research included “interleukin”, “interleukin-10”, “cytokine”, “tuberculosis”, “*Mycobacterium tuberculosis*”, “single nucleotide polymorphism”, “variant”, “genotype”, “mutation”. To minimize potential publication bias, no restrictions were placed on language, sample size, and time period.

Inclusion and Exclusion Criteria

All included studies have to fulfill the following characteristics and inclusion criteria: (a) case-control studies focused on associations between IL-10 -1082G/A, -819T/C, and −592A/C polymorphisms and the risk of TB; (b) the diagnosis of TB should meet the internationally accepted criteria; (c) genotype distribution in both cases and controls were available for estimating an odds ratio (OR) with 95% confidence interval (CI). The exclusion criteria of the meta-analysis were: (a) animal studies; (b) meta-analyses, letters, reviews, meeting abstracts, or editorial comments; (c) studies with duplicate data, incomplete data, and unavailable data. When an individual author published several articles obtained from the same patient population, only the newest or most complete article was included in the analysis.

### Data Extraction

Data were independently abstracted by two reviewers (Liang and Li) using a standard protocol and data-collection according to the inclusion criteria. The following data were collected from each study: first author’s name, year of publication, country, ethnicity, source of controls, sample size, genotyping method, and number of cases and controls for IL-10 −1082G/A, −819T/C, and −592A/C polymorphisms. An attempt was made to contact authors if data presentation was incomplete or if it was necessary to resolve an apparent conflict or inconsistency in the article. Any disagreements were resolved by consensus.

### Statistical Analysis

Review manager 5.0 program provided by the Cochrane Library and Stata (Version12.0, Stata Corporation) were used to perform all the statistical analysis. The combined odds ratio (OR) with its 95% confident interval (CI) was used to assess the strength of the association between the IL-10 polymorphisms and TB risk. The significance of the combined OR was determined by the Z-test, in which *P*<0.05 was considered significant. The pooled ORs were calculated for allele model (mutation [M] allele versus wild [W] allele), dominant model (WM+MM versus WW), recessive model (MM versus WM+WW), homozygote comparison (MM versus WW), and heterozygote comparison (WM versus WW), respectively. Two models of pooling data for dichotomous outcomes were conducted: the random-effects model and the fixed-effects model. Heterogeneity assumption was assessed by the Chisquare based Q test and was regarded to be statistically significant if *P*<0.10. When the *P*≥0.10, the pooled statistical analysis was calculated by the fixed-effects model, otherwise, a random-effect model was used. To evaluate the ethnicity-specific effects, subgroup analyses were performed by ethnic group. The potential publication bias was assessed by Begg’s funnel plot and Egger’s test [14], [15].

## Results

### Characteristics of Studies

**Figure 1:**
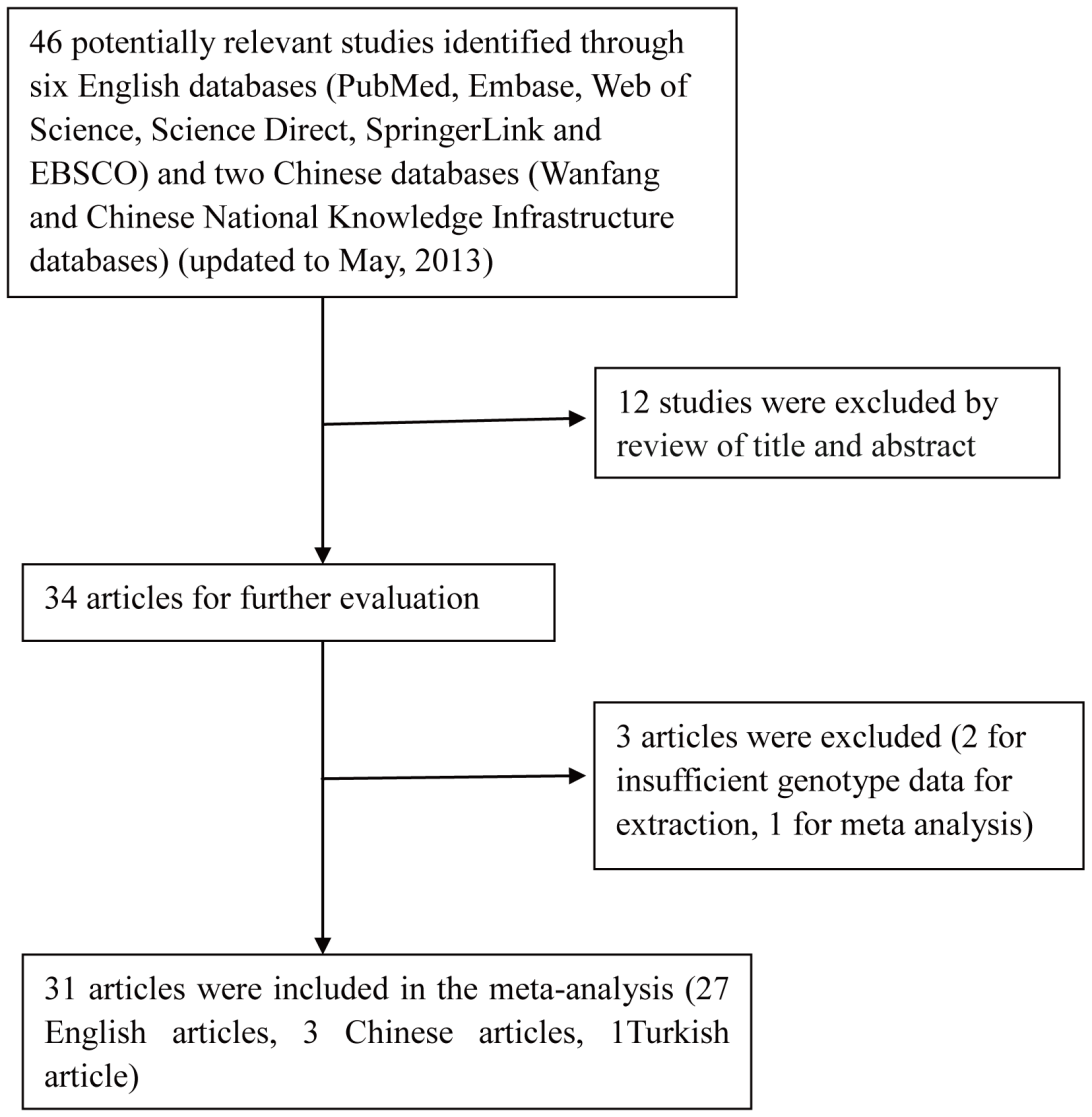
Flow diagram of the literature search and trial selection process.

The flow chart that displays the study selection process was shown in Figure 1. A total of 31 case-control studies, including 6,559 cases and 7,768 controls, were finally identified according to inclusion and exclusion criteria. There are 29 case-control studies concerning -1082G/A polymorphism [16]–[44], 14 case-control studies concerning -819T/C polymorphism [16], [18], [20]–[23], [25], [31], [33], [37]–[39], [42], [45], and 16 case-control studies concerning -592A/C polymorphism [16], [20]–[25], [28], [31], [33], [37]–[40], [45], [46]. Among the 31 eligible studies, 17 of them were of Asians [16], [18], [19], [26], [28], [30]–[32], [34], [36], [39], [41]–[44], [46], 6 studies were of Europeans [17], [21], [25], [27], [35], [37], 5 studies were of Africans [20], [22], [29], [33], [38], and 3 studies were of Americans [23], [24], [40]. Controls were selected from healthy population in all studies and most studies used frequency-matched controls to the cases by age, sex, or ethnicity. The genotype distributions among the controls of all studies were in agreement with HWE except for ten studies for the -1082G/A, two studies for the -819T/C, and three studies for the -592A/C. The detailed characteristics of the eligible studies included in this meta-analysis were shown in Table 1.

**Table 1:** Baseline characteristics of the 31 eligible studies for the analysis of IL-10 polymorphism.

Studies	Year	Country	Ethnicity	Source of controls	Sample size	SNP studied	Genotyping method	HWE
Wu F	2008	China	Asian	HB	61/122	-1082G/A, -819T/C, -592A/C	PCR-RFLP	0.379, 0.125, 0.125
Scola A	2003	Italy	European	PB	45/114	-1082G/A	ARMS-PCR	<0.001
Selvaraj P	2008	India	Asian	PB	155/183	-1082G/A, -819T/C	PCR-RFLP	0.204, 0.174
Sharada RS	2012	India	Asian	HB	104/102	-1082G/A	ARMS-PCR	0.057
Bellamy R	1998	Gambia	African	HB	401/408	-1082G/A, -819T/C, -592A/C	PCR-SSP	0.824, 0.779, 0.779
Oral HB	2006	Turkey	European	HB	81/50	-1082G/A, -819T/C, -592A/C	PCR-SSP	0.060, 0.320, 0.320
Ben-Selma W	2011	Tunisian	African	HB	131/95	-1082G/A, -819T/C, -592A/C	PCR-RFLP	0.020, 0.957, 0.957
Heno MI	2006	Colombia	American	HB	190/135	-1082G/A, -819T/C, -592A/C	PCR-SSP	0.674, 0.410, 0.518
Garcia-Elorriaga G	2006	Mexico	American	HB	77/60	-1082G/A, -592A/C	Taqman	0.728, <0.001
Ates O	2008	Turkey	Asian	HB	128/80	-1082G/A, -819T/C, -592A/C	ARMS-PCR	0.978, 0.819, 0.819
Delgado JC	2002	Cambodian	Asian	HB	356/106	-1082G/A,	PCR-RFLP	<0.001
Ulger M	2013	Turkey	European	HB	84/110	-1082G/A	PCR-RFLP	<0.001
Shin HD	2005	Korea	Asian	HB	449/851	-1082G/A, -592A/C	MAPA	0.168, 0.631,
Mosaad YM	2010	Egypt	African	HB	110/118	-1082G/A	ARMS-PCR	<0.001
Oh JH	2007	Korea	Asian	HB	145/117	-1082G/A	ARMS-PCR	0.612
Liang L	2011	China	Asian	HB	235/78	-1082G/A, -819T/C, -592A/C	SNaPshot assay	0.589, 0.253, 0.253
Ansari A	2009	Pakistan	Asian	PB	178/376	-1082G/A	ARMS-PCR	<0.001
Thye T	2009	Ghana	African	PB	2010/2346	-1082G/A, -819T/C, -592A/C	FRET	0.542, 0.380, 0.551
Ansari A	2011	Pakistan	Asian	PB	102/166	-1082G/A	ARMS-PCR	<0.001
Lopez-Maderueyo D	2003	Spain	European	HB	113/100	-1082G/A	ARMS-PCR	0.949
Meenakshi P	2013	India	Asian	HB	100/100	-1082G/A	PCR-RFLP	0.058
Mhmoud N	2013	Sudan	Asian	HB	191/206	-819T/C, -592A/C	PCR-RFLP	<0.001, <0.001
Mei	2012	China	Asian	HB	169/156	-592A/C	PCR-RFLP	0.622
Ma Z	2007	China	Asian	HB	40/40	-1082G/A	PCR-SSP	0.292
Yang H	2010	China	Asian	HB	198/200	-1082G/A	PCR-SSP	0.253
Liu XX	2009	China	Asian	HB	141/135	-1082G/A, -819T/C	PCR-RFLP	<0.001, <0.001
Trajkov D	2009	Macedonia	European	PB	75/299	-1082G/A, -819T/C, -592A/C	PCR-SSP	<0.001, 0.879, 0.403
Fitness J	2004	Malawi	African	HB	210/705	-1082G/A, -819T/C, -592A/C	ARMS-PCR	0.524, 0.062, 0.035
Amirzargar AA	2006	Iran	Asian	HB	41/123	-1082G/A, -819T/C, -592A/C	PCR-SSP	<0.001, 0.671, 0.671
Taype CA	2010	Peru	American	HB	626/513	-1082G/A, -592A/C	Taqman PCR	0.142, 0.055
Prabhu Anand S	2007	India	Asian	HB	132/143	-1082G/A	PCR-RFLP	0.123

PB, population-based controls, HB, hospital-based controls. HWE, Hardy–Weinberg equilibrium. PCR, polymerase chain reaction; SSP, sequence-specific primers; ARMS, amplification refractory mutation system; RFLP, restriction fragment length polymorphism; FRET, fluorescence resonance energy transfer. MAPA, multiplex automated primer extension analysis.

### Quantitative Data Synthesis

The evaluation of association between IL-10 polymorphisms and TB risk was presented in Table 2.

**Table 2:** Determination of the genetic effects of ***IL-10*** polymorphisms on TB and subgroup analysis.

	Allele model		Dominant model		Recessive model			Homozygous model		Heterozygous model	
	OR [95% CI]	*P*	OR [95% CI]	*P*	OR [95% CI]		*P*	OR [95% CI]	*P*	OR [95% CI]	*P*
**-1082G/A**	**A allele vs. G allele**		**AA+GA vs. GG**		**AA vs. GG+GA**			**AA vs. GG**		**GA vs. GG**	
overall	0.97 [0.79–1.20]	0.81	0.95 [0.68–1.34]	0.79	0.92 [0.75–1.14]		0.46	0.90 [0.61–1.33]	0.59	0.99 [0.72–1.36]	0.96
Asian	1.15 [0.89–1.47]	0.27	1.48 [0.69–3.17]	0.32	1.24 [0.91–1.69]		0.17	1.65 [0.74–3.72]	0.22	1.39 [0.67–2.90]	0.38
European	0.75 [0.56–1.01]	0.05	0.57 [0.37–0.89]	0.01	0.67 [0.36–1.26]		0.21	0.53 [0.26–1.08]	0.08	0.60 [0.39–0.93]	0.02
African	1.13 [0.62–2.07]	0.69	1.03 [0.85–1.25]	0.78	0.76 [0.53–1.09]		0.13	0.84 [0.56–1.24]	0.37	1.09 [0.90–1.32]	0.40
American	0.58 [0.32–1.06]	0.07	0.39 [0.27–0.57]	<0.01	0.53 [0.20–1.41]		0.20	0.31 [0.13–0.77]	0.01	0.46 [0.32–0.68]	<0.01
**-819T/C**	**C allele vs. T allele**		**CC+TC vs. TT**		**CC vs. TC+TT**			**CC vs. TT**		**TC vs. TT**	
overall	0.98 [0.92–1.05]	0.59	1.03 [0.85–1.25]	0.77	0.87 [0.73–1.05]	0.15		0.98 [0.85–1.12]	0.73	1.09 [0.85–1.41]	0.49
Asian	0.83 [0.72–0.96]	0.01	0.94 [0.57–1.54]	0.80	0.60 [0.40–0.90]	0.01		0.63 [0.45–0.88]	0.006	1.10 [0.57–2.10]	0.78
European	1.09 [0.84–1.41]	0.51	1.27 [0.70–2.31]	0.43	1.08 [0.75–1.56]	0.67		1.33 [0.71–2.49]	0.38	1.19 [0.63–2.24]	0.59
African	1.02 [0.94–1.10]	0.62	1.08 [0.94–1.25]	0.27	0.99 [0.88–1.11]	0.88		1.06 [0.90–1.25]	0.46	1.09 [0.93–1.27]	0.29
American	1.00 [0.72–1.38]	1.00	0.89 [0.48–1.64]	0.71	1.06 [0.68–1.66]	0.79		0.94 [0.49–1.81]	0.85	0.85 [0.44–1.63]	0.62
**-592A/C**	**C allele vs. A allele**		**CC+AC vs. AA**		**CC vs. AC+AA**			**CC vs. AA**		**AC vs. AA**	
overall	0.99 [0.83–1.18]	0.90	0.89 [0.74–1.08]	0.25	0.92 [0.78–1.09]	0.32		0.87 [0.68–1.11]	0.27	0.90 [0.76–1.07]	0.24
Asian	0.69 [0.57–0.85]	<0.01	0.65 [0.49–0.85]	0.002	0.62 [0.49–0.79]	<0.01		0.49 [0.34–0.71]	<0.01	0.70 [0.55–0.89]	<0.01
European	1.19 [0.92–1.54]	0.18	1.47 [0.81–2.66]	0.20	1.18 [0.85–1.65]	0.32		1.57 [0.85–2.90]	0.15	1.34 [0.72–2.52]	0.36
African	1.01 [0.92–1.10]	0.90	1.10 [0.94–1.30]	0.24	0.95 [0.83–1.08]	0.45		1.06 [0.88–1.27]	0.54	1.14 [0.96–1.35]	0.14
American	1.56 [1.08–2.27]	0.02	1.04 [0.79–1.36]	0.79	1.24 [0.92–1.69]	0.16		1.26 [0.94–1.69]	0.13	0.86 [0.64–1.15]	0.30

**Figure 2:**
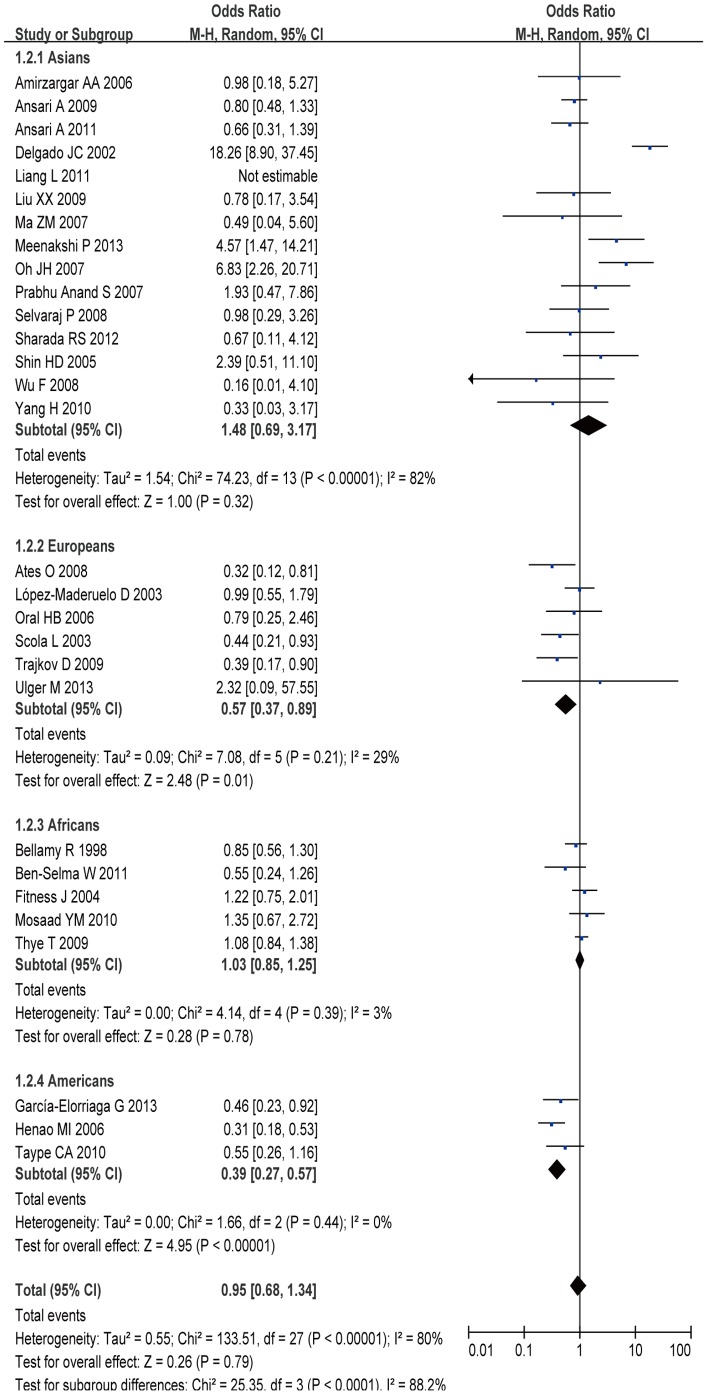
Meta-analysis with a random-effect model for the ORs of tuberculosis risk associated with IL-10 -1082G/A polymorphism in dominant genetic model comparison.

Of the 31 studies investigating the association between IL-10 -1082G/A polymorphism and TB susceptibility, 29 provided enough data to calculate ORs, including 6,199 cases and 7,406 controls. The results of pooling all studies showed that the IL10 -1082 G/A polymorphism was not associated with TB susceptibility in general population under all genetic models (A allele vs. G allele: OR =  0.97, 95% CI = 0.79–1.20, *P* = 0.81; AA+GA vs. GG: OR =  0.95, 95% CI  =  0.68–1.34, *P* = 0.79; AA vs. GA+ GG: OR =  0.92, 95% CI = 0.75–1.14, *P* = 0.46; AA vs. GG: OR =  0.90, 95% CI = 0.61–1.33, *P* = 0.59; GA vs. GG: OR =  0.99, 95% CI = 0.72–1.36, *P* = 0.96) (Figure 2). In the stratified analysis by ethnicity, we found that TB risk was significant decreased in European group under dominant model (Figure 2) (AA+GA vs. GG: OR =  0.57, 95% CI  =  0.37–0.89, *P* = 0.01) and heterozygous model (GA vs. GG: OR =  0.60, 95% CI = 0.39–0.93, *P* = 0.02). However, no significant association between this polymorphism and TB risk was observed in other comparison models in European group. Moreover, significant increased TB risk was observed in dominant model (Figure 2) (AA+GA vs. GG: OR =  0.39, 95% CI  =  0.27–0.57, *P*<0.01), homozygous model (AA vs. GG: OR =  0.31, 95% CI = 0.13–0.77, *P* = 0.01), and heterozygous model (GA vs. GG: OR =  0.46, 95% CI = 0.32–0.68, *P*<0.01) in American group.

**Figure 3:**
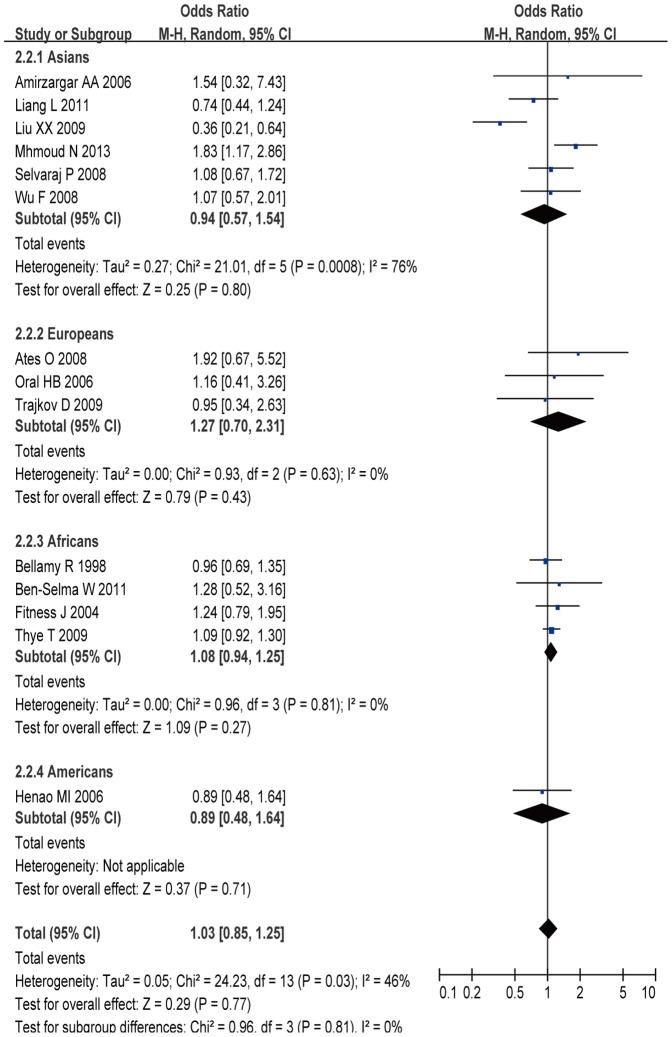
Meta-analysis with a random-effect model for the ORs of tuberculosis risk associated with IL-10 -819T/C polymorphism in dominant genetic model comparison.

A total of 3,584 cases and 4,584 controls from 14 case-control studies were included for data synthesis. The results showed that there was no statistically significant association between IL-10 -819C/T polymorphism and TB risk in general population (C allele vs. T allele: OR =  0.98, 95% CI = 0.92–1.05, *P* = 0.59; CC+TC vs. TT: OR =  1.03, 95% CI  =  0.85–1.25, *P* = 0.77; CC vs. TC+TT: OR =  0.87, 95% CI = 0.73–1.05, *P* = 0.15; CC vs. TT: OR =  0.98, 95% CI = 0.85–1.12, *P* = 0.73; TC vs. TT: OR =  1.09, 95% CI = 0.85–1.41, *P* = 0.49) (Figure 3). In the stratified analyses for the -819C/T polymorphism, there was a significantly decreased risk was observed among Asians in allele model (C allele vs. T allele: OR =  0.83, 95% CI = 0.72–0.96, *P* = 0.01), homozygous model (CC vs. TT: OR =  0.60, 95% CI = 0.40–0.90, *P* = 0.01), and heterozygous model (TC vs. TT: OR =  0.63, 95% CI = 0.45–0.88, *P* = 0.006).

**Figure 4:**
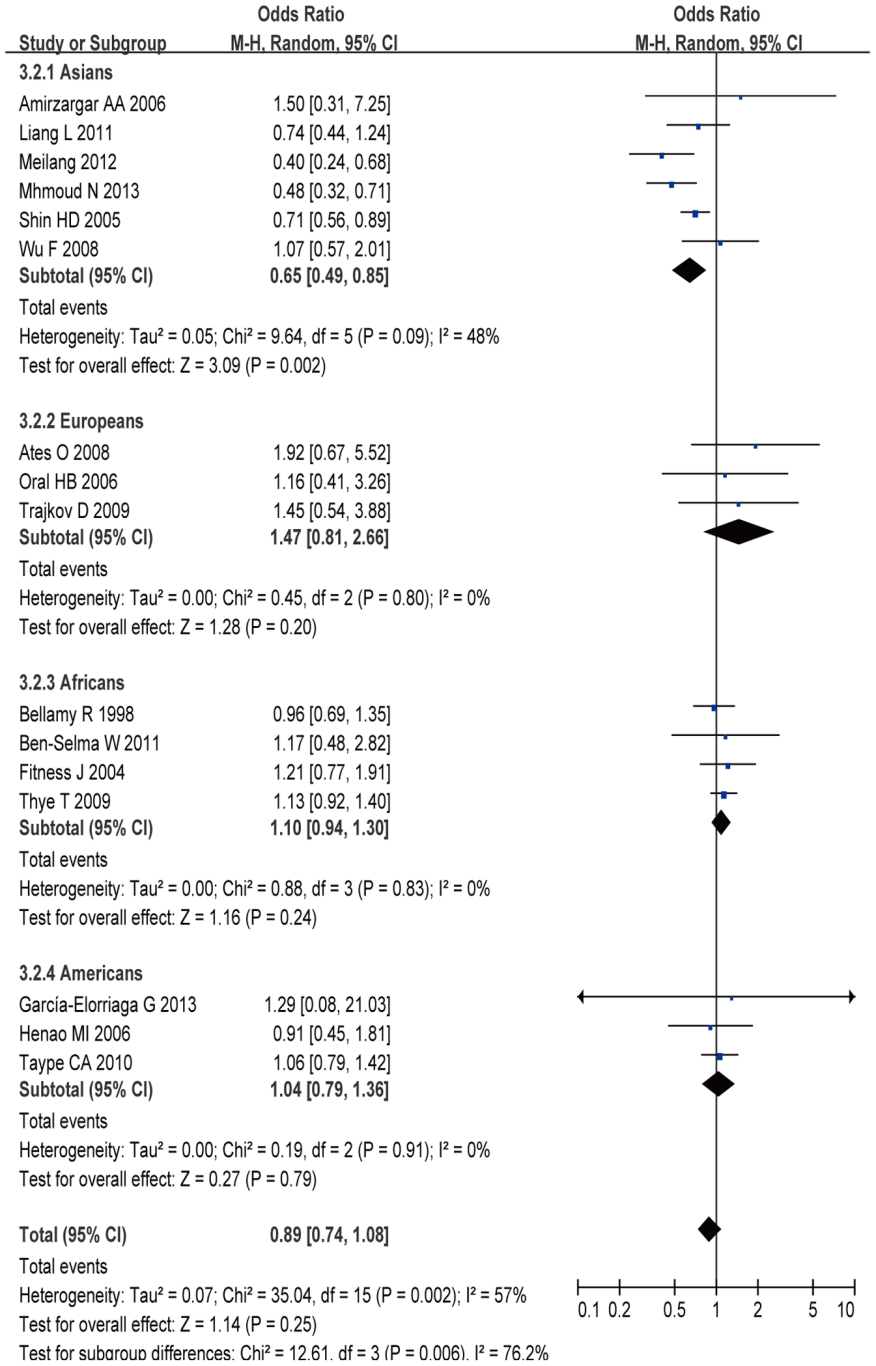
Meta-analysis with a random-effect model for the ORs of tuberculosis risk associated with IL-10 -592A/C polymorphism in dominant genetic model comparison.

A total of 4,063 cases and 5,326 controls from 16 case-control studies were included for data synthesis. In the current meta-analysis, we did not find a significant relationship between IL-10 -592A/C polymorphism and TB risk (C allele vs. A allele: OR =  0.99, 95% CI = 0.83–1.18, *P* = 0.90; CC+AC vs. AA: OR = 0.89, 95% CI  =  0.74–1.08, *P* = 0.25; CC vs. AC+AA: OR =  0.92, 95% CI = 0.78–1.09, *P* = 0.32; CC vs. AA: OR =  0.87, 95% CI = 0.68–1.11, *P* = 0.27; AC vs. AA: OR = 0.90, 95% CI = 0.76–1.07, *P* = 0.24) (Figure 4). In the subgroup analysis by ethnicity, the results indicated that there was significant association between IL-10 -592A/C polymorphism and TB risk in Asians under all gene models (C allele vs. A allele: OR =  0.69, 95% CI = 0.57–0.85, *P*<0.01; CC+AC vs. AA: OR = 0.65, 95% CI  =  0.49–0.85, *P* = 0.002; CC vs. AC+AA: OR =  0.62, 95% CI = 0.49–0.79, *P*<0.01; CC vs. AA: OR =  0.49, 95% CI = 0.34–0.71, *P*<0.01; AC vs. AA: OR = 0.70, 95% CI = 0.55–0.89, *P*<0.01), but not in Europeans, Africans, and Americans, suggesting genetic diversity among ethnicities.

### Publication Bias

**Figure 5:**
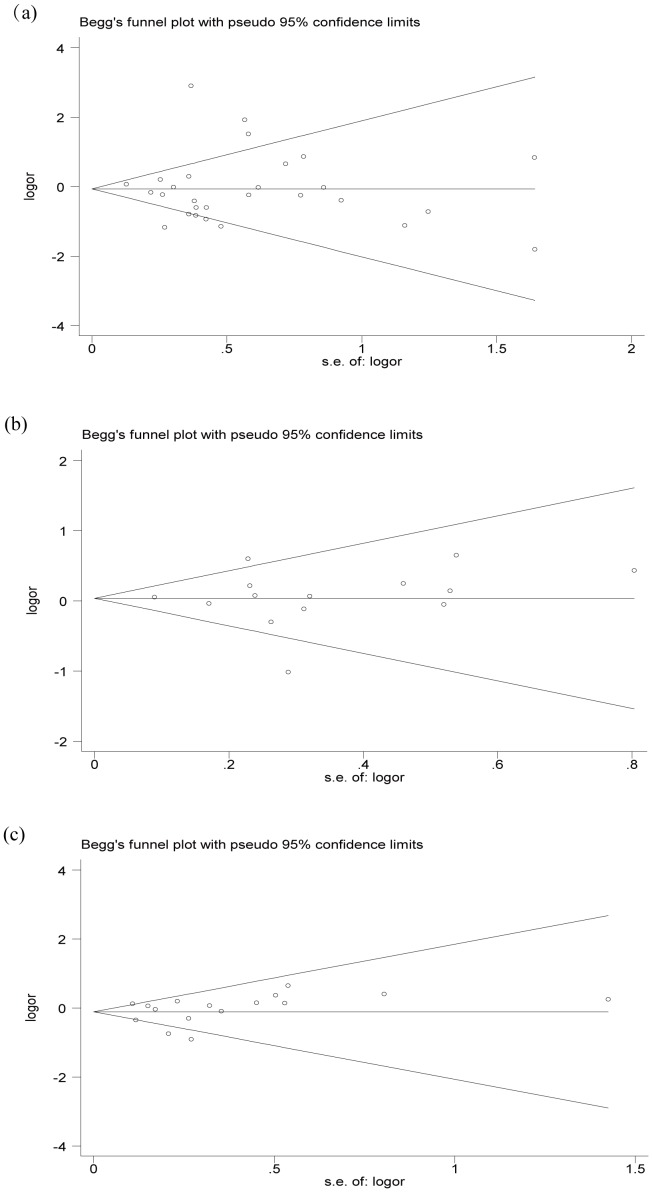
Funnel plot for publication bias of the meta-analysis of tuberculosis risk and IL-10 polymorphisms in dominant genetic model comparison. (a) IL-10 -1082G/A polymorphism, (b) IL-10 -819T/C polymorphism, (c) IL-10 -592A/C polymorphism.

Begg’s funnel plot and Egger’s test were performed to assess the publication bias of included studies. The shapes of the funnel plots did not reveal any evidence of obvious asymmetry under the dominant model (-1082G/A, *P* = 0.953; -819T/C, *P* = 0.661; -592A/C, *P* = 0.685) (Figure. 5). Egger’s test also did not show any significantly statistical evidence of publication bias under the dominant model (-1082G/A, *P* = 0.992; -819T/C, *P* = 0.981; -592A/C, *P* = 0.712), which indicated low risk of publication bias in this meta-analysis.

## Discussion

To date, convincing evidence indicate that the outcome of TB is modulated by the environment as well as bacterial and host genetic components. Many investigations have confirmed that cytokines appear to play the critical roles in the development of TB. Polymorphisms in several cytokine genes have been described and demonstrated to influence gene transcription, leading to interindividual variations in cytokine [47]. IL-10 is a powerful T helper 2 regulatory cytokine and plays an essential role during the latent TB stage, where increased production of this cytokine promotes reactivation of disease in mice [8] and suppression of cell-mediated immunity against the intracellular infection [4]. IL-10 gene is located on the long arm of chromosome 1, where several polymorphisms have been identified within the promoter region, such as −1082G/A, −819T/C, and −592A/C. Genetic studies showed that IL-10 polymorphisms in the promoter region were associated with TB risk, and a number of studies have been performed to investigate that association. However, inconclusive results were obtained. To provide further investigation into these controversial points, a meta-analysis is needed to achieve a more reliable and comprehensive conclusion.

Based on a meta-analysis from 18 studies that contained 4740 cases and 5919 controls, Zhang J, *et al*. found that -819C/T and -592A/C polymorphisms do not affect susceptibility to TB, while the -1082G/A polymorphism was significantly associated with decreased risk of TB only in Europeans [48]. Our meta-analysis, which involved 31 studies including 6559 cases and 7768 controls, also found that the presence of the -1082G/A, -819T/C, and -592A/C genotypes was not associated with the risk of TB in the general population. In our analysis, there was evidence of heterogeneity between studies. It may be due to some factors, including the ethnicity, the selection of methods, definition of cases, and sample sizes.

As recent reports showed that genotype frequencies at IL-10 vary greatly in different populations, particularly in individuals of different ethnicities [49], a subgroup analysis was conducted in our study. Our subgroup analyses showed that the IL-10 -1082G/A genotype significantly decreased TB risk in Europeans and Americans, but not in Asians or Africans. The individuals who carry variant A allele (AA+GA) had a nearly 43% and 63% decreased risk of TB in Europeans and Americans, respectively, suggesting a possible role of ethnic differences in genetic backgrounds and environmental exposures. In stratified analyses for IL-10 -819 T/C, we observed a significant association between IL-10 -819 T/C polymorphism and TB risk in Asians under allele model, recessive model, and homozygous model. We also observed an association between IL-10 -592A/C polymorphism and TB risk in Asians under all gene models. In the case of European population, there were only 4 studies containing 284 cases and 429 controls for IL-10 -819T/C and IL-10 -592A/C polymorphisms analysis. As for American population, there were only one study containing 190 cases and 235 controls for IL-10 -819T/C polymorphism analysis. Therefore, our results should be interpreted with caution, and more case-control studies based on larger sample size of the different ethnicity population should be carried out in the future.

Some limitations of this meta-analysis should be considered when explaining our results. First, given that only published studies were included in the meta-analysis, publication bias may be present, although our results of publication bias showed no significance. Second, some studies were not in agreement with the HWE, making the sample a poor representation. Third, significant between-study heterogeneity was observed in some comparisons, and as such, results may be distorted. Different ethnic populations and different sources of controls may contribute to the heterogeneity. Forth, the interaction of different susceptibility genes and environment factors leaded to the disease, but our study could not assess gene-gene and gene-environment interactions due to the limited information of included studies. Last, but not the least, meta-analysis remains a retrospective research that is subject to the methodological deficiencies of the included studies. In view of these limitations, further studies should focus on the associations of gene polymorphisms and clinical or laboratory characteristic in a large cohort of TB patients.

## Conclusion

In conclusion, despite the several considerations mentioned above, this meta-analysis indicated that three polymorphisms (-1082G/A, -819T/C, and -592A/C) in the IL-10 gene were not associated with the risk of TB in general population. In the subgroup analysis, IL-10 -1082G/A polymorphism was associated with TB risk in Europeans and Americans, and IL-10 -819T/C and -592A/C polymorphisms were significantly associated with TB risk in Asians. In the future, additional large studies are warranted to validate our findings. Future studies should include multi-ethnic groups and use standardized unbiased genotyping methods, and well-matched controls.

## Supporting Information

Checklist S1 PRISMA 2009 Checklist.
(WIZ)
